# Treatment of limb wounds of horses with orf virus IL-10 and VEGF-E accelerates resolution of exuberant granulation tissue, but does not prevent its development

**DOI:** 10.1371/journal.pone.0197223

**Published:** 2018-05-15

**Authors:** Lyn M. Wise, Christa J. Bodaan, Gabriella S. Stuart, Nicola C. Real, Zabeen Lateef, Andrew A. Mercer, Christopher B. Riley, Christine L. Theoret

**Affiliations:** 1 Department of Pharmacology and Toxicology, University of Otago, Dunedin, New Zealand; 2 Department of Microbiology and Immunology, University of Otago, Dunedin, New Zealand; 3 School of Veterinary Science, Massey University, Palmerston North, New Zealand; 4 Comparative Tissue Healing Laboratory, Département de Biomedecine Vétérinaire, Université de Montréal, Montréal, Québec, Canada; Universita degli Studi della Campania Luigi Vanvitelli, ITALY

## Abstract

Bandaging of limb wounds in horses leads to formation of exuberant granulation tissue (EGT) that retards healing due to protracted inflammation, aberrant vascularisation and delayed epithelialisation. EGT is not observed if wounds are left undressed or when wounds are on the body. A previous study showed that short-term administration of proteins derived from orf virus dampened inflammation and promoted epithelialisation of open wounds in horses. Here, we investigated the impact of orf virus interleukin-10 and vascular endothelial growth factor-E on the development and resolution of EGT. Excisional wounds were created on the forelimb of four horses, and bandages were maintained until full healing to induce EGT formation. Matching body wounds were created to ensure EGT was limited to the limb, and to differentiate the effects of the viral proteins on normal healing and on EGT formation. Viral proteins or the hydrogel vehicle control were administered topically to site-matched wounds at day 1, with repeat administration at day 8. Wound healing and EGT formation were monitored macroscopically. Wound margin samples were harvested at 2, 7 and 14 days, and at full healing, with histology used to observe epithelialisation, immunofluorescence used to detect inflammatory cells, angiogenesis and cell death, and qPCR to measure expression of genes regulating inflammation and angiogenesis. Limb wounds developed EGT, and exhibited slower healing than body wounds. Viral protein treatment did not accelerate healing at either location nor limit EGT formation in limb wounds. Treatment of limb wounds did however increase epithelialisation and angiogenesis, without dampening inflammatory cell infiltration or gene expression. The healed wounds also had less occlusion and death of blood vessels and fewer epidermal rete ridges following viral protein treatment. These findings indicate that the viral protein treatment does not suppress wound inflammation or EGT formation, but does promote vascular and epidermal repair and EGT resolution.

## Introduction

Skin wounds in horses occur frequently and are of significant financial and welfare concern to the equine industry [1]. In this species, wounds must often heal by secondary intention because massive tissue loss and considerable contamination preclude primary closure [2]. When wounds occur on the distal extremity of the limb, one of the most common and frustrating complications disturbing repair is a spontaneously developing fibroproliferative disorder known as Exuberant Granulation Tissue (EGT) [2]. The clinical presentation of EGT is strikingly similar to that of human keloid [3] and these conditions share a number of underlying pathophysiologic and histopathologic features [2, 4]. In particular, chronic inflammation [5], aberrant angiogenesis [6–8], and defective wound epithelialisation and contraction [2, 4] are observed.

Although a moist wound healing environment associated with the use of dressings is advocated in other species to manage wounds healing by secondary intention, this approach is not always appropriate in the horse because of its tendency to develop EGT [9–11]. The clinical observation that bandaging can induce excessive wound granulation led to development of an experimental model of equine EGT [12]. The use of an occlusive dressing in this model creates a moist, warm, hypoxic environment that favours angiogenesis [13, 14]. This hypoxic environment also contributes to wound inflammation through hypoxia responsive transcription factor HIF-1α-induced granulocytes and macrophage infiltration and activation [15]. In wounds suffering from EGT, this cellular proliferation physically impedes both wound contraction and migration of keratinocytes over the protruding granulation tissue [5, 16]. It is therefore predicted that treatments that improve wound oxygenation or suppress wound inflammation will dampen EGT formation in this experimental model, thereby improving the timeframe and quality of repair.

Soluble mediators, such as growth factors and cytokines have a critical influence on repair processes. Vascular endothelial growth factors (VEGFs) and interleukin (IL)-10 are two such mediators that exert pleiotropic effects during tissue repair. VEGFs are key regulators of angiogenesis and wound epithelialisation [17]. A viral variant, VEGF-E, when applied to mice, enhanced wound epithelialisation and blood vessel maturation while limiting inflammation [18–21]. IL-10 is an anti-inflammatory cytokine implicated in the scar-free healing of fetal skin wounds [22]. A viral variant, ovIL-10, suppressed pro-inflammatory gene expression and limited the recruitment of monocytes, dendritic cells and mast cells to inflamed murine skin [23, 24], and when applied to wounds, dampened scar tissue formation [24]. Horse skin cells *in vitro* and *in vivo* have been shown to express receptors to ovIL-10 and VEGF-E [25, 26], and to alter inflammatory and angiogenic gene expression in response to the viral proteins. Short-term administration with VEGF-E and ovIL-10 also enhanced epithelialisation and dampened immune cell infiltration of experimentally-induced, undressed, open body and limb wounds in horses [25].

Given the beneficial effects of VEGF-E and ovIL-10 on vascularisation, epithelialisation and inflammation in open wounds, we hypothesised that as a combination therapy these proteins would prevent EGT formation in bandaged wounds on the limbs of horses. We therefore aimed to investigate the effect of the combination therapy in the experimental equine EGT model. Whereas the previous study utilised subcutaneous administration of the proteins, here we predicted that topical administration of the viral proteins would provide more targeted delivery to the wound bed, where they could directly suppress the hypoxia and inflammation that leads to EGT formation, while accelerating wound epithelialization and closure. A hyaluronic acid hydrogel was chosen for delivery due to its proposed biocompatibility [27], regulated release of growth factors and cytokines [28], potential role in fetal skin regeneration [29] and ease of administration in the dressed equine wound. The overall objective of this study was to ascertain if the virus protein-loaded hydrogel was appropriate to control a cutaneous fibroproliferative disorder of horses.

## Materials and methods

### Viral proteins

Recombinant ovIL-10 and VEGF-E were expressed and purified from 293-Epstein-Barr virus Nuclear Antigen-1 cells using FLAG immunoprecipitation, as described previously [30].

### Horses

Four Thoroughbred geldings (ages 5–8 years) were acquired through a racehorse rehoming organisation. They underwent a general physical exam, and were administered tetanus vaccine and broad-spectrum anthelminthics before recruitment into the study. The horses were housed individually on wood shavings, given hay and water *ad libitum*, and had access to a yard for a minimum of one hour per day. During the study, the horses were checked daily for signs of lameness, systemic illness and discomfort using the composite orthopaedic pain scale for horses [31]. At the termination of the study, the animals were adopted by personnel of the School of Veterinary Science or by members of the general public. Ethical approval was granted under protocol number 14/84 by Massey University Animal Ethics Committee.

### Wound model

We used our well-established equine wound model wherein wounds on the body heal rapidly and without complications while wounds on the limb show protracted healing and, when bandaged, develop EGT [12]. The study design is summarised in S1 Fig. Briefly, on day 0 the horses were sedated with detomidine hydrochloride (0.01 mg/kg; intravenous), and pain prevented with the analgesic butorphanol tartrate (0.04 mg/kg; intravenous). Hair was clipped from the dorsolateral surface of the left and right metacarpal areas and the left and right thoracic walls of each horse. Local anaesthesia was performed using 2% lidocaine hydrochloride in an inverted L block on the thoracic wall and a ring block on the proximal metacarpus. Four 1.5 cm X 1.5 cm (2.25 cm^2^) areas on the dorsolateral surface of each metacarpal area beginning just above the metacarpophalangeal joint, and on each lateral thoracic wall, were traced 1.5 cm apart in a staggered vertical column. A full-thickness skin excision was then made of each area, using a scalpel; this skin was kept as the time 0 sample. All wounds were left to heal by second intention. As neither pain nor infection were observed post-surgery in the horses, there was no requirement for administration of local analgesia, non-steroidal anti-inflammatories or antimicrobials during the study.

The limb wounds were bandaged postoperatively for 24 hours to control haemorrhage. Bandaging consisted of non-adherent non-adherent gauze dressing covered by a cotton wool roll held in place with an adhesive tertiary layer. After 24 hours, bandages were removed, the wounds were photographed and then viral proteins were administered to each of the four wounds of one randomly-assigned limb and one thoracic area, in each horse. The dosage and regimen of the viral protein therapy was extrapolated from our previous experiments [25] and was administered topically in a HyStem hydrogel (BioTime, Alameda, CA) vehicle. Hydrogel was prepared according to the manufacturer’s instructions. Equal volumes of the thiol-modified hyaluronan and gelatin were cross-linked by the addition of polyethylene glycol diacrylate, then 0.5 mL was applied to the surface of each wound. For the viral treatment, the proteins were added to the hydrogel components prior to cross-linking, with 20μg VEGF-E and 2μg ovIL-10 administered per wound while for the control wounds, only the hydrogel carrier was administered. Dressings were also used to cover the hydrogel (with / without viral proteins) applied to the wounds on the thoracic area but were removed after 8–12 hours. Conversely, the limb wounds were bandaged for the duration of the study to induce the formation of EGT [12], with bandage changes occurring every 3–4 days to coincide with times at which wound progression was documented photographically. One week after the administration of the viral proteins and hydrogel vehicle, an additional dose of viral proteins (10 μg VEGF-E and 1 μg ovIL-10) was administered by subcutaneous injection to the margin of each of the two wounds in the treated thoracic and metacarpal areas yet to be harvested.

Under the same sedation, analgesia and local anaesthesia conditions used for surgery, full-thickness wound margin samples were harvested with an 8-mm diameter biopsy punch from one wound per site (treated body; control body; treated limb; control limb), beginning with the most distal wound; samples were obtained at 2 days, 7 days, and 14 days after wound creation, and at the time of full healing. These samples were halved at right angle to the junction between the wound and the intact skin border. One half of each sample was incubated in RNAlater stabilisation solution (Thermo Fisher Scientific, Waltham, MA) for 24 hours prior to being stored at -80°C for later RNA isolation. The other half of each sample was positioned with its flat (cut) surface pressed against the bottom of a histology cassette, fixed in neutral-buffered 10% formalin for 7 days and stored in 70% ethanol; these tissue sections were then embedded in paraffin wax. Serial 4μm sections were taken from the fixed blocks orthogonally to the epidermal surface.

### Gross morphology of healing wounds

Time taken for the wound to heal by epithelialisation was noted for each wound. Each wound was photographed with a scale bar to permit correction for distance between camera and the wound. The area was measured in pixels using ImageJ software and corrected to pixel size of scale bar. Wound healing was calculated as percentage of original wound surface area. Macroscopic wound appearance was also scored by two independent observers blind to the treatment regime, as outlined previously [11]. Briefly, protuberance of granulation tissue was scored from 0 (none) to 2 (marked), quality of granulation tissue was scored as 0 (smooth) or 1 (rough), and colour of granulation tissue was determined to be 0 (pink) or 1 (yellow/dark red). The total score with maximum value of 4 was calculated based 50% on protuberance, 25% on quality, and 25% on colour of granulation tissue.

### Quantitative polymerase chain reaction

Skin biopsies were homogenised and total RNA isolated as described previously [25]. Synthesis of cDNA was carried out with total RNA, oligo(dT)15, and random hexamer primers using Superscript III (Invitrogen). Quantitative polymerase chain reactions (qPCR) were performed using Applied Biosystems 7500 Fast PCR machine using PerfeCTa® SYBR® Green FastMix® (Quanta BioSciences) and cDNA equivalent to 5ng RNA. Primer pairs were as previously described [25]. Gene expression was normalised to glyceraldehyde-3-phosphate dehydrogenase (GAPDH) and to gene levels in intact unwounded skin [25, 32].

### Histological analyses

Serial sections from paraffin-embedded tissue samples were stained with Martius Scarlet Blue (MSB) trichrome, with digital photographs taken using an upright microscope (Olympus BX-51). Panoramic images of wound sections were generated using Photoshop (Adobe Systems). Image analyses were performed using ImageJ (National Institutes of Health, Bethesda, MD). Epidermal tongue length was measured from the intact skin into wound tissue along the neo-dermal boundary. Epidermal coverage was calculated as the width of the wound bed covered by neo-epidermis. The thickness of neo-epidermis was calculated as the mean of five measurements equally spanning its length. The number of epidermal protrusions (rete ridges) was counted manually.

### Immunofluorescence analyses

Sections were deparaffinised then rehydrated. Sections underwent antigen retrieval in 10mM sodium citrate (pH 6) at 95°C for 20 min using a decloaking chamber (Biocare), or in tris-buffered saline (TBS, pH 7.4) at 37°C for 20 min. All tissues were blocked with 15% bovine serum albumin (BSA) for 1 hour, with antibody incubations conducted in TBS containing 0.1% BSA and 0.05% Triton-X100. For visualising granulocytes, monocytes and macrophages, sections were incubated with an antibody against calprotectin (MAC387, mouse monoclonal, ab22506, Abcam, 1:200 dilution) overnight at 4°C. The antibody was detected with goat anti-mouse IgG (H+L) AlexaFluor® 488 (A-11001; Invitrogen, 1:500 dilution) after 1 hour incubation at RT. For visualising blood vessels, sections were incubated with antibodies against CD31 (M-20 goat polyclonal, sc-1506; Santa Cruz, 1:200 dilution) and collagen IV (rabbit polyclonal, ab6586; Abcam, 1:50 dilution) with sequential overnight incubations at 4°C. The antibodies were then detected with donkey anti-goat immunoglobulin G (H+L) AlexaFluor® 594 (A-11058; Invitrogen, 1:500 dilution) and goat anti-rabbit IgG (H+L) AlexaFluor® 488 (A-11070; Invitrogen, 1:500 dilution) after 1 hour incubation at room temperature (RT). Nuclei were counter-stained with 75 nM 4’,6-diamidino-2-phenylindole (DAPI, D3571; Invitrogen) for 30 min at RT. For visualising apoptotic and necrotic cells, the Dead End Fluorometric TUNEL System (PMG3250; Promega) was used according to manufacturer’s instructions. Slides were mounted with SlowFade Gold anti-fade reagent (S36936; Invitrogen) then visualised using an upright fluorescent microscope (Olympus BX-51). Digital photos were taken of the entire section using CellSens (Olympus) then the images were merged and converted into panoramic images using Photoshop.

The number of inflammatory cells in the granulation tissue and adjacent intact skin were quantitated in images taken of calprotectin-stained sections. Nucleated green-stained cells were counted using ImageJ particle analysis. The corresponding region was outlined using the freehand tool in ImageJ, the outlined area measured, then cell number expressed relative to that area. Endothelial cells in the granulation tissue were quantitated in images taken of CD31 and collagen IV-stained sections. Images were converted to RGB stacks then the granulation tissue outlined as described above. The threshold in the red channel was adjusted automatically to highlight the CD31-stained cells, and then measurements were taken as a percentage of total granulation tissue area. The threshold in the green channel was adjusted automatically to highlight the collagen IV-stained blood vessels which were then counted using ImageJ particle analysis. The corresponding region was measured and vessel number relative to that area was calculated. The area of the unstained lumen within each collagen IV-stained blood vessel was also obtained by the ImageJ particle analysis. As erythrocytes decrease in size to less than 3 μm to traverse the smallest capillaries, only blood vessels with a cross-sectional area of less than 7.07 μm^2^ were deemed to be occluded. [8, 33]. The percentage of blood vessels identified using ImageJ particle analysis with lumen areas less than 7 μm^2^ was calculated, with occlusion of each vessel confirmed manually. Dead cells within the granulation tissue were quantitated in images taken of TUNEL-stained sections. Again, images were converted to RGB stacks and thresholds applied in the green channel to highlight the TUNEL-stained cells (area of one cell defined as < 50μm^2^) and blood vessels (area of three adjacent cells defined as > 150μm^2^). The number of TUNEL^+ve^ cells and blood vessels within the granulation tissue were counted using ImageJ particle analysis and expressed relative to that area.

### Statistical analyses

For wound surface area analyses and EGT scores, a linear mixed model with *a priori* contrasts followed by a Benjamini-Hochberg sequential adjustment was performed with SAS v9.4, as previously described [25]. For gene expression, histological and immunofluorescence analyses, ratio paired *t*-tests (two-tailed) were conducted for matched treatments, sites and times. The level of statistical significance was defined as *p* ≤ 0.05.

## Results

### The viral protein treatment does not macroscopically improve healing or limit EGT formation in limb wounds

**Fig 1:**
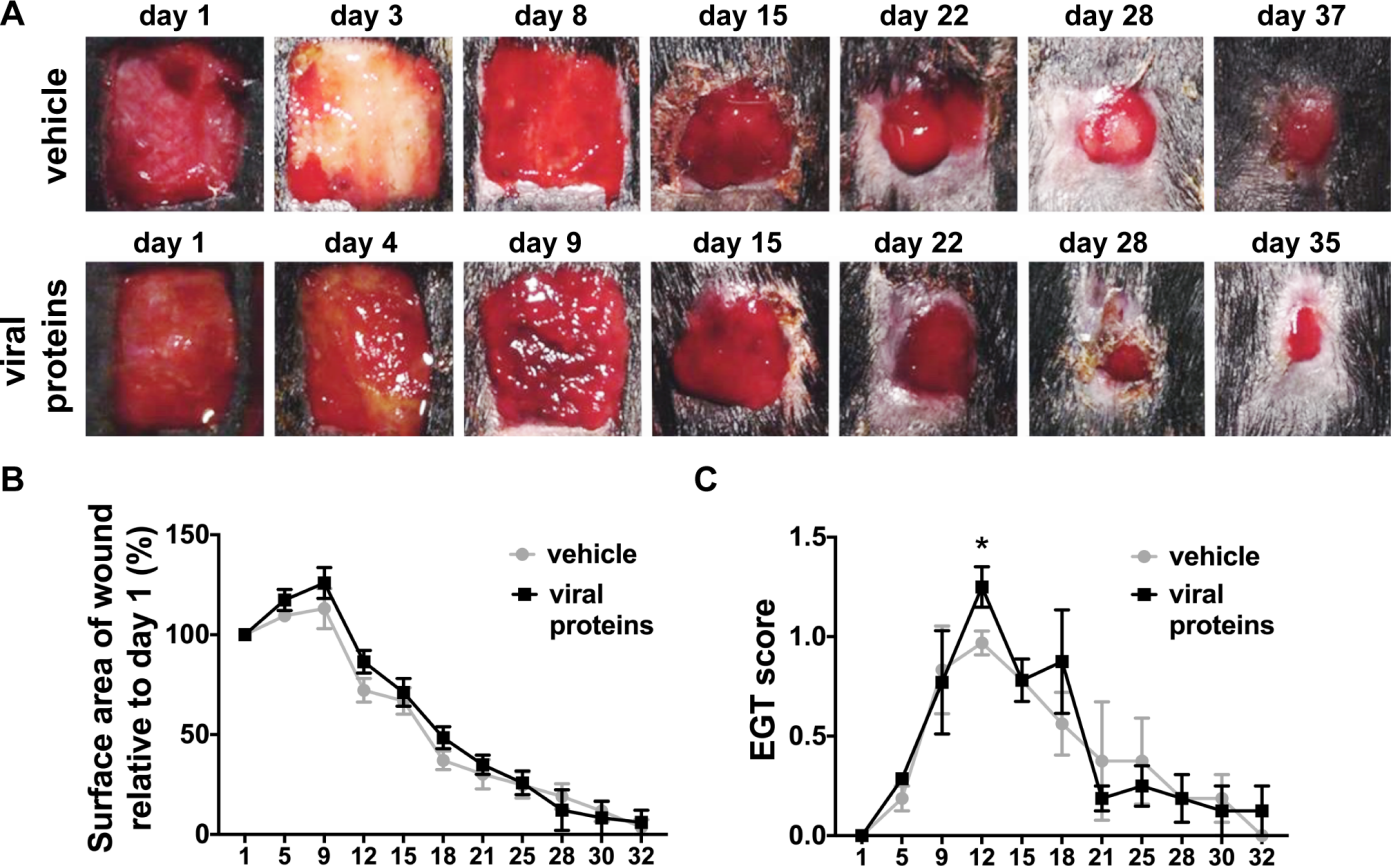
Healing of limb wounds of horses following treatment with viral proteins. (A) Representative photos of healing limb wounds following a single topical administration of vehicle (HyStem hydrogel) or viral proteins in vehicle (VEGF-E and ovIL-10) prior to bandaging on day 1 and a subcutaneous injection of vehicle (saline) or viral proteins in vehicle at day 8. (B) Surface area and (C) exuberant granulation tissue (EGT) formation of healing wounds at the days indicated. Wound surface area is calculated relative to the original wound area. EGT formation was scored on 50% on protuberance (0 none– 2 marked), 25% colour (0 pink– 1 yellow-red) and 25% on quality (0 smooth—1 rough). Values represent mean ± SEM, n = 4. *p ≤ 0.05 between means of viral protein treated and vehicle control wounds at each time point, as determined using a linear mixed model with *a priori* contrasts followed by a Benjamini-Hochberg sequential adjustment.

We trialled a viral protein therapy combining ovIL-10 with VEGF-E that had previously been shown to have transient benefits to healing of equine wounds. In an attempt to dampen EGT formation the viral proteins were administered topically to the wounds in a hydrogel vehicle then covered with dressing. Difficulties with retaining the dressing on the body meant that these wounds had less than 12 hr exposure to the viral protein and vehicle. The dressing however successfully remained on the limb wounds protecting the viral protein and vehicle. As previously observed with this model, bandaged limb wounds took longer to heal than body wounds (S2 Fig). The limb wounds also showed increased EGT formation, peaking at day 12, and persisting until day 25 (S2 Fig). As limb wounds showed no immediate visual improvement in wound closure or reduction in EGT formation after topical administration of the viral proteins, after 1 week, an additional dose (10 μg VEGF-E and 1 μg ovIL-10, or saline control) was administered subcutaneously at the margin of each limb wound (Fig 1A). No significant differences in healing kinetics were however observed between treated and control wounds of the limb at any time-point (Fig 1B). EGT formation in limb wounds was for the most part grossly unaffected by viral protein treatment, with the exception of day 12 when the EGT score of treated wounds was significantly greater than that of control wounds (Fig 1C, *p* < 0.0001). As expected with the limited retention of the vehicle, healing kinetics did not differ between treated and control body wounds (not shown). Further analyses therefore focused on the effect of treatment on healing processes in limb wounds only.

### The viral protein treatment increases epidermal repair and resolution in limb wounds

**Fig 2:**
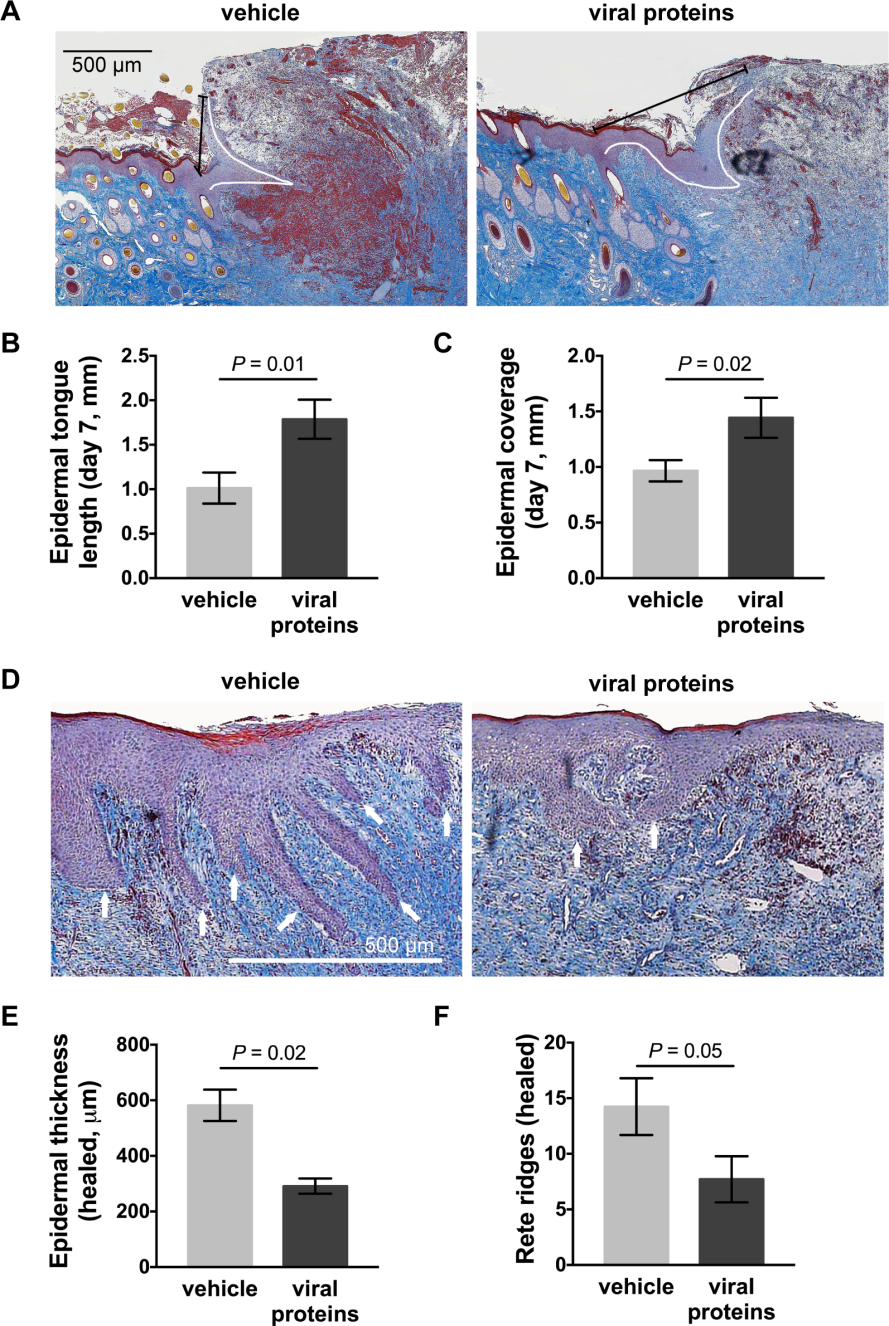
Epithelialisation of limb wounds of horses following treatment with viral proteins. (A) Representative photos of wound margin sections taken 7 days post-wounding, stained with MSB trichrome. White and black lines in each photo indicate the length of the epidermal tongue and epidermal coverage, respectively. (B) Epidermal tongue length and (C) epidermal coverage following topical administration of vehicle or viral proteins. (D) Representative photos of healed wounds stained with MSB trichrome. Rete ridges are indicated by white arrows. (E) Epidermal thickness (measured at 5 equidistant points across the neo-epidermis) and (F) number of epidermal rete ridges projecting into the neo-dermis of healed wounds following administration of vehicle or viral proteins. Values represent mean ± SEM, n = 4. *P* values indicated were determined using a two-tailed ratio paired *t*-test.

MSB trichrome-stained sections from both control and treated limb wounds showed ballooning granulation tissue with extensive fibrin deposits and epidermal hyperplasia at the wound edge at day 7 (Fig 2A). Topical treatment with the viral proteins increased the length of the epidermal tongue and increased epidermal coverage of the granulation tissue (Fig 2A–2C, length: *p =* 0.01, coverage: *p =* 0.02). The beneficial effects on epithelialisation observed following treatment were no longer evident at day 14 (not shown). The neo-epidermis of healed control wounds was substantially thicker than that of intact skin, with extensive rete ridging projecting into the dermal tissue (Fig 2D). Treatment with the viral proteins significantly decreased both the epidermal thickness (Fig 2E, *p* = 0.02) and the number of rete ridges in the healed wounds (Fig 2F, *p* = 0.05).

### The viral protein treatment had varied effects on the inflammatory response in healing limb wounds

**Fig 3:**
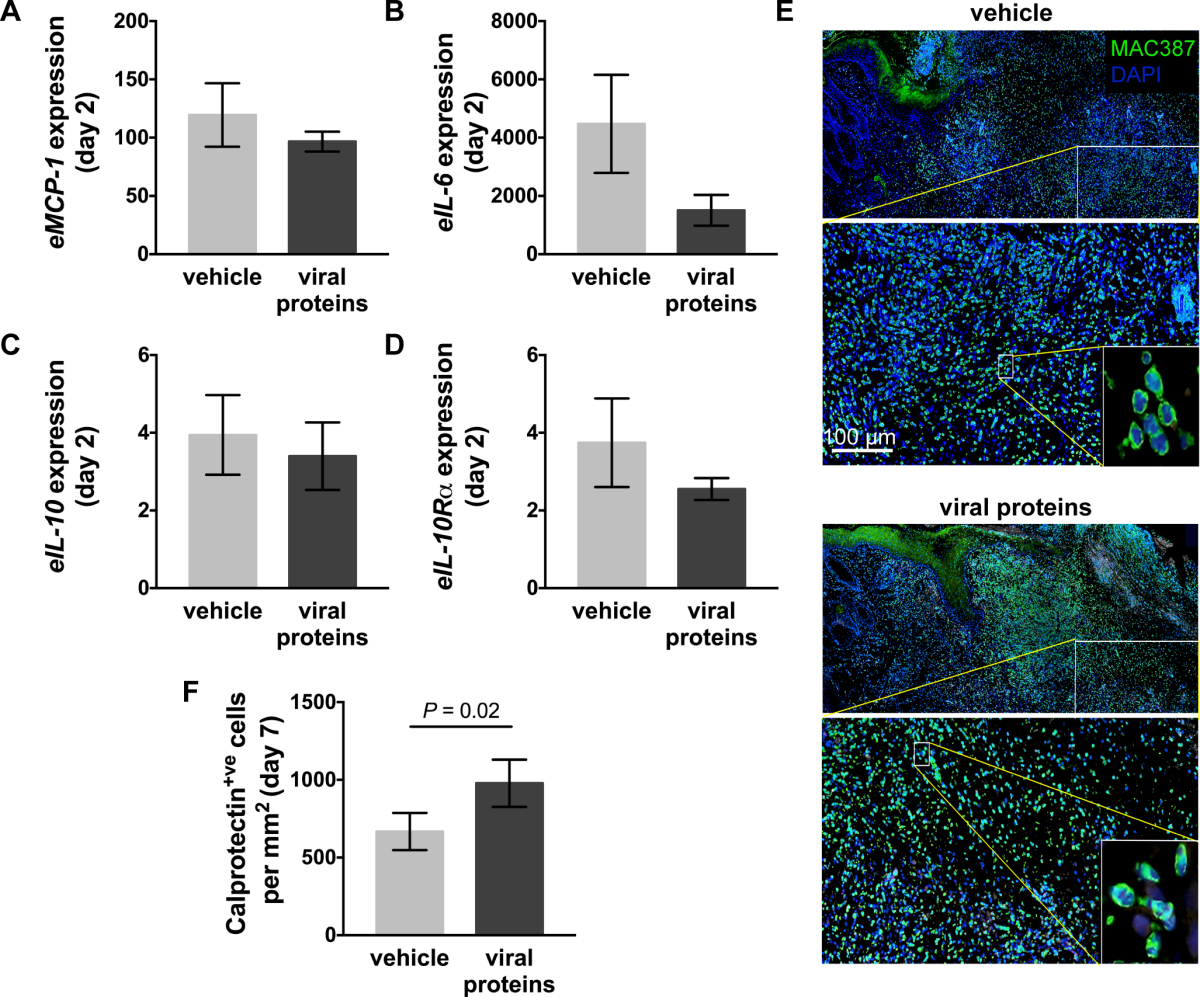
Inflammation in limb wounds of horses following treatment with viral proteins. Quantitative PCR was used to measure the expression of (A) equine (e)*MCP-1*, (B) *eIL-6*, (C) *eIL-10*, and (D) *eIL-10Rα* in wound margin samples taken 2 days after wounding. Expression of mRNA is relative to that of GAPDH and to levels measured in unwounded skin (day 0). (E) Representative photos of skin sections taken 7 days post-wounding stained with DAPI (blue) and an antibody against the inflammatory cell marker calprotectin (MAC387: green). Enlarged images show nucleated inflammatory cells. (F) Number of calprotectin^+ve^ cells in the granulation tissue and surrounding skin of wounds following administration of vehicle or viral proteins. Values represent mean ± SEM, n = 4. *P* values indicated were determined using a two-tailed ratio paired *t*-test.

As observed previously, creation of wounds on the limb lead to a substantial increase in expression of selected pro-inflammatory genes, and to a limited extent anti-inflammatory genes, at day 2 relative to baseline values in intact skin (Fig 3A–3D). Treatment with the viral proteins did not however significantly impact expression of pro-inflammatory chemokines and cytokines, *eMCP-1* or *eIL-6* (Fig 3A and 3B), or the anti-inflammatory cytokine, *eIL-10* or its receptor *eIL-10Rα* (Fig 3C and 3D). Consistent with this, no changes in cytokine or chemokine expression were identified at other time points examined (not shown). Inflammatory gene expression leads to recruitment of inflammatory cells such as granulocytes, monocytes and tissue macrophages. A fluorescent antibody against calprotectin was therefore used to detect these cells in the limb wounds. At day 7, large numbers of inflammatory cells were found in the intact skin adjacent to the wound and dispersed throughout the granulation tissue (Fig 3E). Quantitation of the stained cells showed there was an increase within treated limb wounds at day 7 (Fig 3F, *p* = 0.02). At later time points examined, the number of calprotectin^+ve^ cells observed in the limb wounds had substantially reduced and did not differ between treated and control wounds (not shown).

### The viral protein treatment increases functional blood vessel formation in healing limb wounds

**Fig 4:**
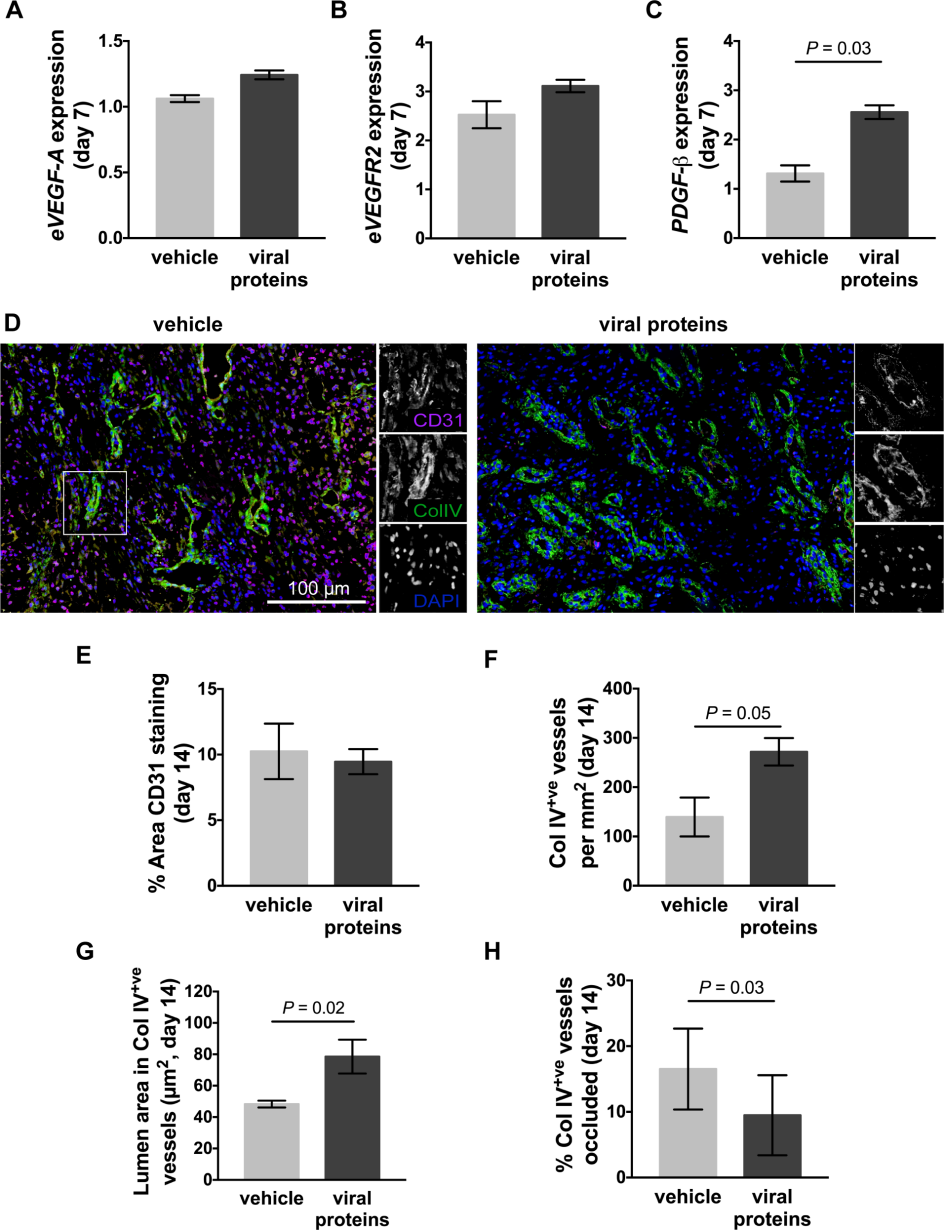
Vascularisation of limb wounds of horses following treatment with viral proteins. Quantitative PCR was used to measure the expression of (A) *eVEGF-A*, (B) *eVEGFR2*, and (C) *ePDGF-*β in wound margin samples taken 7 days after wounding. Expression of mRNA is relative to that of GAPDH and to levels measured in unwounded skin (day 0). (D) Representative photos of skin sections taken 14 days post-wounding, stained with DAPI (blue) and antibodies against blood vessel endothelial cells (CD31: pink) and basal lamina (collagen IV: green). Enlargements show single colour images of representative blood vessels. (E) Area of CD31 staining, (F) number of collagen IV^+ve^ blood vessels, (G) lumen area in collagen IV^+ve^ blood vessels and (H) percentage of blood vessels occluded (lumen area less than 7 μm^2^) in the granulation tissue of wounds following administration of vehicle or viral proteins. Values represent mean ± SEM, n = 4. *P* values indicated were determined using a two-tailed ratio paired *t*-test.

Impaired healing has previously been associated with aberrant levels of angiogenic gene expression. In contrast, very little change in the expression of the endothelial cell mitogen *eVEGF-A* or its receptor *eVEGFR-2*, or the pericyte mitogen *ePDGF-β* was observed at day 7 relative to that of intact skin (Fig 4A–4C). Treatment with the viral proteins did not alter expression of, *eVEGF-A* or *eVEGFR-2* (Fig 4A and 4B), but did significantly increase expression of *ePDGF-β* (Fig 4C, *p* = 0.03). No further changes in angiogenic gene expression were identified at other time points examined (not shown). To examine differences in vascularisation between wounds, an antibody against CD31 was used to detect platelet endothelial cell adhesion molecule (PECAM)-1 on endothelial cells, and an antibody against collagen (col) IV was used to detect the basal lamina of mature blood vessels. At day 14, following both the topical and subcutaneous treatment, a large number of CD31^+ve^ endothelial cells were observed in the granulation tissue of limb wounds, with individual cells showing higher intensity staining than those associated with col IV stained blood vessels (Fig 4D). In treated limb wounds, the intensity of CD31 staining was lower than that of control wounds and was predominantly associated with the col IV stained blood vessels (Fig 4D). It also appeared that the lumen of blood vessels in the treated wounds were larger than that of control wounds (Fig 4D). There were equivalent numbers of CD31^+ve^ cells in treated and control limb wounds at day 14 (Fig 4E), but the number of col IV^+ve^ blood vessels in the granulation tissue had significantly increased (Fig 4F, *p* = 0.05). There was also a significant increase in the area of the lumen within the col IV^+ve^ blood vessels of wounds treated with the viral proteins when compared to control wounds (Fig 4G, *p* = 0.02). As equine red blood cells traffic through lumen of greater than 7 μm^2^, the proportion of blood vessels within the granulation tissue with lumen inferior to that area was quantitated. There were significantly less blood vessels with lumen diameters inferior to 7 μm^2^ in treated limb wounds than in that of controls (Fig 4H, *p* = 0.03).

### The increase in blood vessel diameter following viral protein treatment is associated with reduced blood vessel death in healed limb wounds

**Fig 5:**
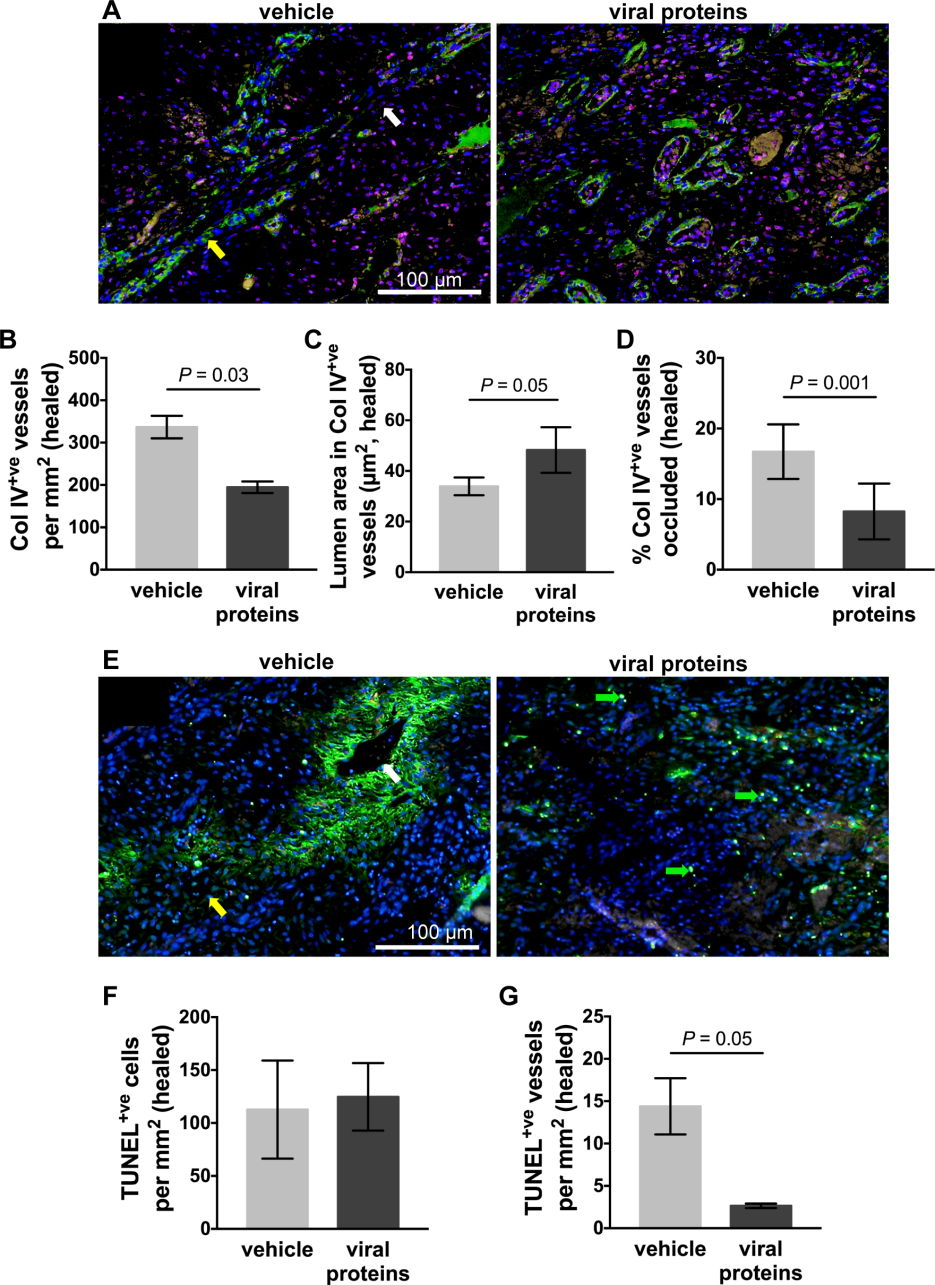
Blood vessel integrity in healed limb wounds of horses following treatment with viral proteins. (A) Representative photos of healed wounds stained with DAPI (blue) and antibodies against blood vessel endothelial cells (CD31: pink) and basal lamina (collagen IV: green). (B) Number of collagen IV^+ve^ blood vessels (C) lumen area in collagen IV^+ve^ blood vessels and (D) percentage of blood vessels occluded in the scar tissue of healed wounds following administration of vehicle or viral proteins. (E) Representative photos of healed wounds stained with DAPI (blue) and by TdT dUTP Nick-End Labelling (TUNEL: green). Green arrows indicate individual dying cells. Yellow and white arrows indicate a healthy and a dying blood vessel, respectively. Number of TUNEL^+ve^ (F) cells (area of single cell < 50 μm^2^) and (G) blood vessels (area of 3 or more adjacent cells > 150 μm^2^) in the scar tissue of healed wounds following administration of vehicle or viral proteins. Values represent mean ± SEM, n = 4. *P* values indicated were determined using a two-tailed ratio paired *t*-test.

To assess whether the changes in blood vessel diameter were maintained, vascularisation was again examined in the healed limb wounds. In control wounds, a greater association was observed between CD31^+ve^ endothelial cells and col IV^+ve^ blood vessels at the time of healing (Fig 5A) than observed at day 14. Although a greater number of blood vessels were observed in the healed control wounds, the lumen of the blood vessels was very narrow and many vessels showed areas of reduced CD31 and col IV staining intensity (Fig 5A). Relative to control wounds, the healed scar tissue of treated wounds had a significant reduction in the number and lumen area of the col IV^+ve^ blood vessel (Fig 5A–5C, vessels: *p* = 0.03, lumen area: *p* = 0.05). There were also significantly fewer occluded blood vessels with lumen diameters less than 7 μm^2^ in treated limb wounds than in control wounds (Fig 5D, *p* = 0.001). To assess if the loss of blood vessel staining was associated with cell death, TUNEL was used to detect apoptotic and necrotic cells in the healed scar tissue (Fig 5E). Individual stained cells were observed in both control and treated wounds (Fig 5E), but larger areas of staining corresponding to regions of low intensity CD31 and col IV blood vessel staining were observed in the control wounds (Fig 5A). Quantitation of individual stained cells showed that the level of cell death was equivalent in treated and control wounds (Fig 5F). Quantitation of stained regions (with a combined area of greater than 3 cells) associated with blood vessels, however, showed a substantial reduction in treated wounds (Fig 5G, *p* = 0.05).

## Discussion

This study set out to examine whether topical treatment of equine wounds with a combination of a viral anti-inflammatory cytokine and growth factor could prevent development of EGT. An established model was used in which excisional wounds on the limbs (metacarpal area) of horses were bandaged so as to induce EGT formation. We observed an initial delay in healing of limbs wound compared to body wounds, that coincided with peak EGT formation 12 days after wounding. As previously observed [4, 5], histological sections from limb wounds showed ballooning EGT formation, along with extensive epidermal thickening and rete ridge formation. The healing limb wounds also showed abundant pro-inflammatory cytokine gene expression and inflammatory cell infiltrate, consistent with the pronounced inflammatory response previously reported [5]. Angiogenesis in the limb wounds was also exasperated initially, with newly formed vessels being tortuous in nature, but substantial occlusion and death of vessels was observed in the healed wound. This was consistent with reports of microvessel occlusion and endothelial cell hypertrophy in EGT lesions [7, 8]. The defective vascular response in EGT lesions was not however associated with over-expression of proangiogenic genes (*eVEGF-A* or *ePDGF-β)*. These findings validate the use of this model for evaluating the viral protein combination as a therapy for EGT.

Previous studies showed treatment of chronic wounds in horses with the combination of orf virus VEGF-E and ovIL-10 dampened inflammatory cytokine production and reduced recruitment of immune cells into damaged tissue [25]. These anti-inflammatory effects were attributed to the ovIL-10, which regulated these processes when applied to inflamed or wounded skin in mice [23, 24]. In the current study, however, treatment with these factors did not consistently reduce cytokine production and in fact led to an increase in inflammatory cells in the healing wound. There are a few possible explanations for the difference in efficacy of the ovIL-10. In this study the viral factors were initially applied using a topical hyaluronic acid gel as a vehicle, while in previous studies the ovIL-10 was applied by subcutaneous or intradermal injection [23–25]. In this study, topical application, which requires no prior sedation, was chosen due to its ease of administration for veterinary clinicians, clients and patients. Human or murine IL-10 treatment of skin wounds has also shown efficacy when applied by intradermal or subcutaneous injection or by transgenic over-expression [34–36], but topical delivery has not been reported. It is therefore possible that IL-10 fails to elicit its anti-inflammatory effects when applied topically, and that it should be administered more deeply into the tissues. Although the viral factors were applied subcutaneously after the start of EGT formation in this study, no change in inflammatory gene expression or infiltrate was observed, which suggest this treatment may have been too late to prevent induction of the inflammatory response.

Hydrogels have not previously been used to deliver anti-inflammatory cytokines to skin wounds. As the gel used in this study remained on the wound surface for only a few days it is possible that insufficient IL-10 was released into the wound to mediate any anti-inflammatory effects. Previous studies have however shown that only brief exposure to IL-10 is necessary to dampen wound inflammation and scarring [35]. Direct delivery into the wound fluids may have led to proteolytic degradation and failure of the ovIL-10 to penetrate the tissue at sufficient levels. Another possibility is that the hyaluronic acid gel impacted wound inflammation such that it negated the effects of the ovIL-10. The hydrogel consists of high molecular weight hyaluronic acid, which is reportedly anti-inflammatory [37, 38]. It is however likely that the hydrogel is degraded by hyaluronidases within the wound [39], resulting in production of low molecular weight by-products that enhance inflammatory processes [37, 38, 40]. The hyaluronic acid gel has not previously been tested in wounded skin, so its degradation and impact on wound inflammation should be evaluated further. In antigen-induced rheumatoid arthritis, treatment with hyaluronic acid led to biphasic effects, with initial inhibition of acute inflammation followed by delayed promotion of chronic inflammation [41]. This suggests that the hyaluronic acid-based hydrogel may not be suitable for delivery of anti-inflammatory cytokines.

In previous studies, subcutaneous treatment of equine limb wounds with the combination of VEGF-E and ovIL-10 led to a transient increase in epithelialisation [25]. This was attributed to the viral VEGF-E, which directly promotes keratinocyte proliferation and migration and indirectly enhances matrix metalloproteinase expression [18]. In the current study, topical treatment with the viral factors also enhanced epithelialisation of EGT lesions, but this did not translate to enhanced closure of limb wounds. Topical administration of recombinant human VEGF-A has proven successful in accelerating wound healing in rodents and humans [42, 43], but accelerated healing was achieved when treatment persisted until epithelialisation was complete [18, 42, 43]. It is therefore likely that the brief hydrogel treatment used in this study was insufficient, and that longer-term topical administration with VEGF-E may have greater benefit. In the current study, a reduction in epidermal hyperplasia and rete ridge formation was also observed in the healed tissue following the topical and repeat subcutaneous treatment. Whether the effects of VEGF-E on epidermal resolution can be attributed to the route of delivery is unclear, but the findings do suggest that repeat administration is beneficial.

VEGF-E has been shown to directly induce endothelial cell proliferation and migration leading to angiogenesis in intact and wounded skin [30, 44, 45]. Consistent with this, an increase in the number of blood vessels within the granulation tissue of limb wounds of horses was observed following treatment with the viral factors. In the previous studies, subcutaneous administration of the viral factors previously failed to induce angiogenesis in chronic limb wounds of horses [25], which suggests that the topical administration or delayed repeat injection used here was beneficial. Both delivery routes have previously shown efficacy with VEGF-E [18] and VEGF-A [42, 43]. In the current study, a substantial reduction in the number of blood vessels was also observed in the healed wounds following the viral protein treatment. Typically, blood vessels resolve once tissue has healed, and remodelling into scar tissue begins [46]. This suggests that treated equine wounds initiated remodelling quicker than control wounds.

Epidermal remodelling has also been linked to blood vessel remodelling, with rete ridges projecting precisely between capillary loops of the dermal papillae [47]. Evidence suggests this epidermal architecture may be dependent on pericytes, their vascular coverage or the signalling environment they maintain [48–50]. In treated limb wounds of horses, fewer occluded and dying blood vessels were observed, which suggests that the viral factors improve blood vessel functionality in a manner likely to relieve hypoxia. Anti-angiogenic factors have been implicated in vascular remodelling [51], with thrombospondin 2, secreted protein acidic and rich in cysteine *(*SPARC) and pigment epithelium-derived factor reportedly under expressed in limb wounds of horses [52]. Expression of these factors was not quantified in the current study, but the viral protein treatment did increase expression of *ePDGF*, which enhances pericyte coverage of newly formed blood vessels, thereby inhibiting endothelial cell proliferation and improving capillary stability [53]. Pericytes also contribute to the loss of microvasculature during remodelling through secretion of the anti-angiogenic CXCR3 ligands, IP-9 (CXCL11) and IP-10 (CXCL10)[54]. VEGF-E increases PDGF gene expression and pericyte coverage of blood vessels in murine wounds [19], but further analyses are needed to define the pathways whereby it may enhance blood vessel functionality, and potentially epidermal remodelling.

Fibroproiferative disorders of the skin are also a significant burden for humans, with keloids occurring in response to trauma in 15–20% of individuals of Hispanic, African and Asian ancestory [55]. While there is evidence of genetic susceptibility, the underlying pathogenesis of keloids is ill-defined. Animal models are essential to understanding this disorder and improving therapeutic outcomes. The majority of wound healing research conducted to has been done in rodents, however the concordance between results of wound healing therapies tested in mice and rats then trialed in human patients is only 53%, raising the possibility that findings of rodent-based studies may not translate to improved clinical outcomes [56]. The wound repair processes in horses, however, shares many similarities with that in humans. As with humans, limb wounds in horses heal majoritarily through epithelialisation rather than by contraction, with genetics contributing to the spontaneous development of fibroproliferative disorders [2, 3]. The underlying pathogenesis of keloids is thought to result from endothelial dysfunction, leading to chronic inflammation in the injured dermal tissues [57]. As reported in human keloids [58], a dysregulated cytokine profile is associated with EGT formation [5, 7], as observed in this study. Hypoxia, endothelial cell hypertrophy and microvessel occlusion, have been reported in both equine EGT [7, 52, 59] and human keloids [60–62], consistent with findings in this study. As observed in this study, EGT lesions exhibit marked epidermal proliferation and rete ridge formation [63], features that have also been reported in keloids [64]. While many parallels exist, relative to keloids, EGT lesions show an increased polymorphonuclear cell infiltrate, scant myofibroblasts, and an absence of ‘keloidal’ collagen [63]. This is in contrast to keloids which continue to produce a dense collagen matrix that extends beyond the wound margin and continues to grow for years [55]. The management of keloids, as with EGT, remains a challenge, with limited evidence as to effective therapeutic regimens [55]. With keloids, prevention and treatment measures focus mainly on lesion removal through scar revision surgery or cryo-, radio- or laser-therapy, or on the reduction of inflammation through administration of corticosteroids or 5-Fluorouracil [65]. For treatment of EGT, excision is still accepted as the best approach [66], while the application of corticosteroids to arrest EGT formation is controversial due to their reported negative effects on wound angiogenesis, contraction, epithelialisation and closure [67, 68]. Silicone sheet dressings have, however, been shown to prevent recurrence of EGT in horses [11], which is in accordance with scar prevention studies in humans, in which silicone gel or sheeting applied after full healing reduced the incidence of hypertrophic scarring in at risk individuals [69, 70]. These numerous consistencies support the use of equine EGT as a model of human keloids, and suggest that findings from this study may have be relevant and have clinical applicability for humans.

## Conclusions

This study further highlights the similarities between EGT and human keloid, with dressed equine limb wounds suffering from excess inflammation, vascular impairments and epidermal hyperplasia. Short-term administration of the viral factors, VEGF-E and ovIL-10, using a topical hydrogel, transiently increased wound vascularisation and epithelialisation, but failed to suppress inflammation and EGT formation. The viral protein treatment did however have lasting effects on epidermal and vascular resolution. These findings suggest that when delivered topically, the ovIL-10 may have reduced efficacy as an anti-inflammatory, while VEGF-E retains its previously reported effects on repair and remodelling processes. The findings also suggest that inflammation may be the key driver of EGT formation since relief of vascular impairments only enhanced EGT resolution. Future studies aimed at preventing EGT development in horses or potentially keloid scarring in man, should investigate effective routes of delivery for anti-inflammatories, such as the ovIL-10. In the absence of inflammation, the necessity, and timing of administration for topical treatments such as VEGF-E that target epidermal or vascular remodelling should then be re-evaluated. Balanced administration, or induction, of pro-and anti-angiogenic factors will be critical, so as to promote wound repair and resolution without exasperating cutaneous fibroproliferation.

## Supporting information

S1 Fig Schematic of study design. On day 0, each horse received four 1.5 cm X 1.5 cm (2.25 cm^2^) full-thickness skin wounds. After 24 hours, the wounds were administered hydrogel with or without the viral proteins (20μg VEGF-E and 2μg ovIL-10) to each of the four wounds of one randomly-assigned limb and one thoracic area. Limb wounds were bandaged for the duration of the study, while thoracic wounds were dressed following treatment for only 8–12 hours. After 8 days, the two wounds yet to be harvested received an additional treatment of saline with or without the viral proteins (10 μg VEGF-E and 1 μg ovIL-10) by subcutaneous injection. Biopsies were taken from one wound per site (treated body; control body; treated limb; control limb), in the order indicated (1, 2, 3, 4), on day 2, 7, 14 and once the wound had healed.
(TIF)

S2 Fig Healing of body and limb wounds of horses. (A) Representative photos of healing wounds on the body (top row) or limb (bottom row) taken at the days indicated. (B) Surface area and (C) exuberant granulation tissue (EGT) formation of healing wounds at the days indicated. Wound surface area is calculated relative to the original wound area. EGT formation was scored 50% on protuberance (0 none– 2 marked), 25% on colour (0 pink– 1 yellow-red) and 25% on quality (0 smooth—1 rough). Values represent mean ± SEM, n = 4.
(TIF)

## References

[1] Theoret CL, Bolwell CF, Riley CB. A cross-sectional survey on wounds in horses in New Zealand. N Z Vet J. 2016;64(2):90–4. doi: 10.1080/00480169.2015.1091396 26357976 10.1080/00480169.2015.1091396

[2] Theoret CL, Wilmink JM. Aberrant wound healing in the horse: naturally occurring conditions reminiscent of those observed in man. Wound Repair Regen. 2013;21(3):365–71. doi: 10.1111/wrr.12018 23441750 10.1111/wrr.12018

[3] Ud-Din S, Volk SW, Bayat A. Regenerative healing, scar-free healing and scar formation across the species: current concepts and future perspectives. Exp Dermatol. 2014;23(9):615–9. doi: 10.1111/exd.12457 24863070 10.1111/exd.12457

[4] Theoret CL, Olutoye OO, Parnell LK, Hicks J. Equine exuberant granulation tissue and human keloids: a comparative histopathologic study. Vet Surg. 2013;42(7):783–9. doi: 10.1111/j.1532-950X.2013.12055.x 24015864 10.1111/j.1532-950X.2013.12055.x

[5] Wilmink JM, van Weeren PR, Stolk PW, Van Mil FN, Barneveld A. Differences in second-intention wound healing between horses and ponies: histological aspects. Equine Vet J. 1999;31(1):61–7. 9952331 10.1111/j.2042-3306.1999.tb03792.x

[6] Celeste CJ, Deschesne K, Riley CB, Theoret CL. Skin temperature during cutaneous wound healing in an equine model of cutaneous fibroproliferative disorder: kinetics and anatomic-site differences. Vet Surg. 2013;42(2):147–53. doi: 10.1111/j.1532-950X.2012.00966.x 22742866 10.1111/j.1532-950X.2012.00966.x

[7] Dubuc V, Lepault E, Theoret CL. Endothelial cell hypertrophy is associated with microvascular occlusion in horse wounds. Can J Vet Res. 2006;70(3):206–10. 16850943 PMC1477938

[8] Lepault E, Celeste C, Dore M, Martineau D, Theoret CL. Comparative study on microvascular occlusion and apoptosis in body and limb wounds in the horse. Wound Repair Regen. 2005;13(5):520–9. doi: 10.1111/j.1067-1927.2005.00073.x 16176461 10.1111/j.1067-1927.2005.00073.x

[9] Berry DB 2nd, Sullins KE. Effects of topical application of antimicrobials and bandaging on healing and granulation tissue formation in wounds of the distal aspect of the limbs in horses. Am J Vet Res. 2003;64(1):88–92. 12518884 10.2460/ajvr.2003.64.88

[10] Dart AJ, Perkins NR, Dart CM, Jeffcott LB, Canfield P. Effect of bandaging on second intention healing of wounds of the distal limb in horses. Aust Vet J. 2009;87(6):215–8. doi: 10.1111/j.1751-0813.2009.00428.x 19489777 10.1111/j.1751-0813.2009.00428.x

[11] Ducharme-Desjarlais M, Celeste CJ, Lepault E, Theoret CL. Effect of a silicone-containing dressing on exuberant granulation tissue formation and wound repair in horses. Am J Vet Res. 2005;66(7):1133–9. 16111150 10.2460/ajvr.2005.66.1133

[12] Theoret CL, Barber SM, Moyana TN, Gordon JR. Preliminary observations on expression of transforming growth factors beta1 and beta3 in equine full-thickness skin wounds healing normally or with exuberant granulation tissue. Vet Surg. 2002;31(3):266–73. 11994855 10.1053/jvet.2002.32394

[13] Howard RD, Stashak TS, Baxter GM. Evaluation of Occlusive Dressings for Management of Full-Thickness Excisional Wounds on the Distal Portion of the Limbs of Horses. Am J Vet Res. 1993;54(12):2150–4. 8116952

[14] Knighton DR, Silver IA, Hunt TK. Regulation of wound-healing angiogenesis-effect of oxygen gradients and inspired oxygen concentration. Surgery. 1981;90(2):262–70. 6166996

[15] Cramer T, Yamanishi Y, Clausen BE, Forster I, Pawlinski R, Mackman N, et al HIF-1 alpha is essential for myeloid cell-mediated inflammation. Cell. 2003;112(5):645–57. 12628185 10.1016/s0092-8674(03)00154-5 PMC4480774

[16] Shakespeare V, Shakespeare P. Effects of Granulation-Tissue-Conditioned Medium on the Growth of Human Keratinocytes Invitro. Brit J Plast Surg. 1991;44(3):219–23. 2025760 10.1016/0007-1226(91)90131-3

[17] Johnson KE, Wilgus TA. Vascular Endothelial Growth Factor and Angiogenesis in the Regulation of Cutaneous Wound Repair. Adv Wound Care. 2014;3(10):647–61. 10.1089/wound.2013.0517 PMC4183920 25302139

[18] Wise LM, Inder MK, Real NC, Stuart GS, Fleming SB, Mercer AA. The vascular endothelial growth factor (VEGF)-E encoded by orf virus regulates keratinocyte proliferation and migration and promotes epidermal regeneration. Cell Microbiol. 2012;14(9):1376–90. doi: 10.1111/j.1462-5822.2012.01802.x 22507661 10.1111/j.1462-5822.2012.01802.x

[19] Wise LM, Stuart GS, Real NC, Fleming SB, Mercer AA. VEGF Receptor-2 Activation Mediated by VEGF-E Limits Scar Tissue Formation Following Cutaneous Injury. Adv Wound Care. 2017:In press. 10.1089/wound.2016.0721 PMC6080089 30087804

[20] Zheng Y, Murakami M, Takahashi H, Yamauchi M, Kiba A, Yamaguchi S, et al Chimeric VEGF-E(NZ7)/PlGF promotes angiogenesis via VEGFR-2 without significant enhancement of vascular permeability and inflammation. Arterioscler Thromb Vasc Biol. 2006;26(9):2019–26. doi: 10.1161/01.ATV.0000233336.53574.a1 16794222 10.1161/01.ATV.0000233336.53574.a1

[21] Zheng Y, Watanabe M, Kuraishi T, Hattori S, Kai C, Shibuya M. Chimeric VEGF-ENZ7/PlGF specifically binding to VEGFR-2 accelerates skin wound healing via enhancement of neovascularization. Arterioscler Thromb Vasc Biol. 2007;27(3):503–11. doi: 10.1161/01.ATV.0000256459.06671.3c 17194893 10.1161/01.ATV.0000256459.06671.3c

[22] King A, Balaji S, Le LD, Crombleholme TM, Keswani SG. Regenerative Wound Healing: The Role of Interleukin-10. Adv Wound Care. 2014;3(4):315–23. 10.1089/wound.2013.0461 PMC3985521 24757588

[23] Bennett JR, Lateef Z, Fleming SB, Mercer AA, Wise LM. Orf virus IL-10 reduces monocyte, dendritic cell and mast cell recruitment to inflamed skin. Virus Res. 2016;213:230–7. doi: 10.1016/j.virusres.2015.12.015 26732486 10.1016/j.virusres.2015.12.015

[24] Wise LM, Stuart GS, Real NC, Fleming SB, Mercer AA. Orf virus IL-10 accelerates wound healing while limiting inflammation and scarring. Wound Repair Regen. 2014;22(3):356–67. doi: 10.1111/wrr.12169 24844335 10.1111/wrr.12169

[25] Bodaan CJ, Wise LM, Wakelin KA, Stuart GS, Real NC, Mercer AA, et al Short-term treatment of equine wounds with orf virus IL-10 and VEGF-E dampens inflammation and promotes repair processes without accelerating closure. Wound Repair Regen. 2016;24(6):966–80. doi: 10.1111/wrr.12488 27681311 10.1111/wrr.12488

[26] Wakelin KA, Wise LM, Bodaan CJ, Mercer AA, Riley CB, Theoret CL. Orf virus interleukin-10 and vascular endothelial growth factor-E modulate gene expression in cultured equine dermal fibroblasts. Vet Dermatol. 2016;27(5):434–8. doi: 10.1111/vde.12370 27550846 10.1111/vde.12370

[27] Serban MA, Scott A, Prestwich GD. Use of hyaluronan-derived hydrogels for three-dimensional cell culture and tumor xenografts. Curr Protoc Cell Biol. 2008;Chapter 10:Unit 10 4. 10.1002/0471143030.cb1014s40 PMC3319462 18819087

[28] Hosack LW, Firpo MA, Scott JA, Prestwich GD, Peattie RA. Microvascular maturity elicited in tissue treated with cytokine-loaded hyaluronan-based hydrogels. Biomaterials. 2008;29(15):2336–47. doi: 10.1016/j.biomaterials.2008.01.033 18313745 10.1016/j.biomaterials.2008.01.033 PMC2387277

[29] Balaji S, King A, Marsh E, LeSaint M, Bhattacharya SS, Han N, et al The role of interleukin-10 and hyaluronan in murine fetal fibroblast function in vitro: implications for recapitulating fetal regenerative wound healing. PLoS One. 2015;10(5):e0124302 doi: 10.1371/journal.pone.0124302 25951109 10.1371/journal.pone.0124302 PMC4423847

[30] Wise LM, Ueda N, Dryden NH, Fleming SB, Caesar C, Roufail S, et al Viral vascular endothelial growth factors vary extensively in amino acid sequence, receptor-binding specificities, and the ability to induce vascular permeability yet are uniformly active mitogens. J Biol Chem. 2003;278(39):38004–14. doi: 10.1074/jbc.M301194200 12867434 10.1074/jbc.M301194200

[31] Bussieres G, Jacques C, Lainay O, Beauchamp G, Leblond A, Cadore JL, et al Development of a composite orthopaedic pain scale in horses. Res Vet Sci. 2008;85(2):294–306. doi: 10.1016/j.rvsc.2007.10.011 18061637 10.1016/j.rvsc.2007.10.011

[32] Deschene K, Celeste C, Boerboom D, Theoret CL. Constitutive expression of hypoxia-inducible factor-1 alpha in keratinocytes during the repair of skin wounds in horses. Wound Repair Regen. 2011;19(2):250–9. doi: 10.1111/j.1524-475X.2010.00663.x 21362093 10.1111/j.1524-475X.2010.00663.x

[33] Smith JE, Mohandas N, Shohet SB. Variability in erythrocyte deformability among various mammals. Am J Physiol. 1979;236(5):H725–30. doi: 10.1152/ajpheart.1979.236.5.H725 443394 10.1152/ajpheart.1979.236.5.H725

[34] Gordon A, Kozin ED, Keswani SG, Vaikunth SS, Katz AB, Zoltick PW, et al Permissive environment in postnatal wounds induced by adenoviral-mediated overexpression of the anti-inflammatory cytokine interleukin-10 prevents scar formation. Wound Repair Regen. 2008;16(1):70–9. doi: 10.1111/j.1524-475X.2007.00326.x 18086289 10.1111/j.1524-475X.2007.00326.x

[35] Kieran I, Knock A, Bush J, So K, Metcalfe A, Hobson R, et al Interleukin-10 reduces scar formation in both animal and human cutaneous wounds: results of two preclinical and phase II randomized control studies. Wound Repair Regen. 2013;21(3):428–36. doi: 10.1111/wrr.12043 23627460 10.1111/wrr.12043

[36] Peranteau WH, Zhang L, Muvarak N, Badillo AT, Radu A, Zoltick PW, et al IL-10 overexpression decreases inflammatory mediators and promotes regenerative healing in an adult model of scar formation. J Invest Dermatol. 2008;128(7):1852–60. doi: 10.1038/sj.jid.5701232 18200061 10.1038/sj.jid.5701232

[37] Litwiniuk M, Krejner A, Speyrer MS, Gauto AR, Grzela T. Hyaluronic Acid in Inflammation and Tissue Regeneration. Wounds. 2016;28(3):78–88. 26978861

[38] Petrey AC, de la Motte CA. Hyaluronan, a crucial regulator of inflammation. Front Immunol. 2014;5:101 doi: 10.3389/fimmu.2014.00101 24653726 10.3389/fimmu.2014.00101 PMC3949149

[39] Fronza M, Caetano GF, Leite MN, Bitencourt CS, Paula-Silva FW, Andrade TA, et al Hyaluronidase modulates inflammatory response and accelerates the cutaneous wound healing. PLoS One. 2014;9(11):e112297 doi: 10.1371/journal.pone.0112297 25393024 10.1371/journal.pone.0112297 PMC4230982

[40] Kavasi RM, Berdiaki A, Spyridaki I, Corsini E, Tsatsakis A, Tzanakakis G, et al HA metabolism in skin homeostasis and inflammatory disease. Food Chem Toxicol. 2017;101:128–38. doi: 10.1016/j.fct.2017.01.012 28109952 10.1016/j.fct.2017.01.012

[41] Roth A, Mollenhauer J, Wagner A, Fuhrmann R, Straub A, Venbrocks RA, et al Intra-articular injections of high-molecular-weight hyaluronic acid have biphasic effects on joint inflammation and destruction in rat antigen-induced arthritis. Arthritis Res Ther. 2005;7(3):R677–86. doi: 10.1186/ar1725 15899053 10.1186/ar1725 PMC1174961

[42] Galeano M, Deodato B, Altavilla D, Cucinotta D, Arsic N, Marini H, et al Adeno-associated viral vector-mediated human vascular endothelial growth factor gene transfer stimulates angiogenesis and wound healing in the genetically diabetic mouse. Diabetologia. 2003;46(4):546–55. doi: 10.1007/s00125-003-1064-1 12677400 10.1007/s00125-003-1064-1

[43] Hanft JR, Pollak RA, Barbul A, van Gils C, Kwon PS, Gray SM, et al Phase I trial on the safety of topical rhVEGF on chronic neuropathic diabetic foot ulcers. J Wound Care. 2008;17(1):30–2, 4–7. doi: 10.12968/jowc.2008.17.1.27917 18210954 10.12968/jowc.2008.17.1.27917

[44] Wise LM, Savory LJ, Dryden NH, Whelan EM, Fleming SB, Mercer AA. Major amino acid sequence variants of viral vascular endothelial growth factor are functionally equivalent during Orf virus infection of sheep skin. Virus Res. 2007;128(1–2):115–25. doi: 10.1016/j.virusres.2007.04.018 17524510 10.1016/j.virusres.2007.04.018

[45] Wise LM, Veikkola T, Mercer AA, Savory LJ, Fleming SB, Caesar C, et al Vascular endothelial growth factor (VEGF)-like protein from orf virus NZ2 binds to VEGFR2 and neuropilin-1. Proc Natl Acad Sci U S A. 1999;96(6):3071–6. 10077638 10.1073/pnas.96.6.3071 PMC15896

[46] Wietecha MS, Cerny WL, DiPietro LA. Mechanisms of vessel regression: toward an understanding of the resolution of angiogenesis. Curr Top Microbiol Immunol. 2013;367:3–32. doi: 10.1007/82_2012_287 23224648 10.1007/82_2012_287

[47] Lawlor KT, Kaur P. Dermal Contributions to Human Interfollicular Epidermal Architecture and Self-Renewal. Int J Mol Sci. 2015;16(12):28098–107. doi: 10.3390/ijms161226078 26602926 10.3390/ijms161226078 PMC4691026

[48] Helmbold P, Lautenschlager C, Marsch W, Nayak RC. Detection of a physiological juvenile phase and the central role of pericytes in human dermal microvascular aging. J Invest Dermatol. 2006;126(6):1419–21. doi: 10.1038/sj.jid.5700275 16557234 10.1038/sj.jid.5700275

[49] Paquet-Fifield S, Schluter H, Li A, Aitken T, Gangatirkar P, Blashki D, et al A role for pericytes as microenvironmental regulators of human skin tissue regeneration. J Clin Invest. 2009;119(9):2795–806. doi: 10.1172/JCI38535 19652362 10.1172/JCI38535 PMC2735900

[50] Supp DM, Wilson-Landy K, Boyce ST. Human dermal microvascular endothelial cells form vascular analogs in cultured skin substitutes after grafting to athymic mice. FASEB J. 2002;16(8):797–804. doi: 10.1096/fj.01-0868com 12039861 10.1096/fj.01-0868com PMC1820617

[51] DiPietro LA. Angiogenesis and wound repair: when enough is enough. J Leukoc Biol. 2016;100(5):979–84. doi: 10.1189/jlb.4MR0316-102R 27406995 10.1189/jlb.4MR0316-102R PMC6608066

[52] Celeste CJ, Deschene K, Riley CB, Theoret CL. Regional differences in wound oxygenation during normal healing in an equine model of cutaneous fibroproliferative disorder. Wound Repair Regen. 2011;19(1):89–97. doi: 10.1111/j.1524-475X.2010.00639.x 20955347 10.1111/j.1524-475X.2010.00639.x

[53] Mills SJ, Cowin AJ, Kaur P. Pericytes, mesenchymal stem cells and the wound healing process. Cells. 2013;2(3):621–34. doi: 10.3390/cells2030621 24709801 10.3390/cells2030621 PMC3972668

[54] Yates CC, Krishna P, Whaley D, Bodnar R, Turner T, Wells A. Lack of CXC chemokine receptor 3 signaling leads to hypertrophic and hypercellular scarring. Am J Pathol. 2010;176(4):1743–55. doi: 10.2353/ajpath.2010.090564 20203286 10.2353/ajpath.2010.090564 PMC2843466

[55] Gauglitz GG, Korting HC, Pavicic T, Ruzicka T, Jeschke MG. Hypertrophic scarring and keloids: pathomechanisms and current and emerging treatment strategies. Mol Med. 2011;17(1–2):113–25. doi: 10.2119/molmed.2009.00153 20927486 10.2119/molmed.2009.00153 PMC3022978

[56] Sullivan TP, Eaglstein WH, Davis SC, Mertz P. The pig as a model for human wound healing. Wound Repair and Regeneration. 2001;9(2):66–76. 11350644 10.1046/j.1524-475x.2001.00066.x

[57] Ogawa R. Keloid and Hypertrophic Scars Are the Result of Chronic Inflammation in the Reticular Dermis. Int J Mol Sci. 2017;18(3). 10.3390/ijms18030606 PMC5372622 28287424

[58] Jumper N, Hodgkinson T, Paus R, Bayat A. Site-specific gene expression profiling as a novel strategy for unravelling keloid disease pathobiology. PLoS One. 2017;12(3):e0172955 doi: 10.1371/journal.pone.0172955 28257480 10.1371/journal.pone.0172955 PMC5336271

[59] Sorensen MA, Petersen LJ, Bundgaard L, Toft N, Jacobsen S. Regional disturbances in blood flow and metabolism in equine limb wound healing with formation of exuberant granulation tissue. Wound Repair and Regeneration. 2014;22(5):647–53. doi: 10.1111/wrr.12207 24935817 10.1111/wrr.12207

[60] Kischer CW. The microvessels in hypertrophic scars, keloids and related lesions: a review. J Submicrosc Cytol Pathol. 1992;24(2):281–96. 1600518

[61] Ma X, Chen J, Xu B, Long X, Qin H, Zhao RC, et al Keloid-derived keratinocytes acquire a fibroblast-like appearance and an enhanced invasive capacity in a hypoxic microenvironment in vitro. Int J Mol Med. 2015;35(5):1246–56. doi: 10.3892/ijmm.2015.2135 25777304 10.3892/ijmm.2015.2135 PMC4380122

[62] Zhang Q, Oh CK, Messadi DV, Duong HS, Kelly AP, Soo C, et al Hypoxia-induced HIF-1 alpha accumulation is augmented in a co-culture of keloid fibroblasts and human mast cells: involvement of ERK1/2 and PI-3K/Akt. Exp Cell Res. 2006;312(2):145–55. doi: 10.1016/j.yexcr.2005.10.006 16289155 10.1016/j.yexcr.2005.10.006

[63] Theoret CL, Olutoye OO, Parnell LKS, Hicks J. Equine Exuberant Granulation Tissue and Human Keloids: A Comparative Histopathologic Study. Veterinary Surgery. 2013;42(7):783–9. doi: 10.1111/j.1532-950X.2013.12055.x 24015864 10.1111/j.1532-950X.2013.12055.x

[64] Limandjaja GC, van den Broek LJ, Waaijman T, van Veen HA, Everts V, Monstrey S, et al Increased epidermal thickness and abnormal epidermal differentiation in keloid scars. Br J Dermatol. 2017;176(1):116–26. doi: 10.1111/bjd.14844 27377288 10.1111/bjd.14844

[65] Lee HJ, Jang YJ. Recent Understandings of Biology, Prophylaxis and Treatment Strategies for Hypertrophic Scars and Keloids. Int J Mol Sci. 2018;19(3). 10.3390/ijms19030711 PMC5877572 29498630

[66] Theoret C, Schumacher J. Equine wound management Third edition ed. Ames, Iowa, USA: Wiley Blackwell; 2017. x, 550 pages p.

[67] Beer HD, Fassler R, Werner S. Glucocorticoid-regulated gene expression during cutaneous wound repair. Vitam Horm. 2000;59:217–39. 10714241 10.1016/s0083-6729(00)59008-6

[68] Hashimoto I, Nakanishi H, Shono Y, Toda M, Tsuda H, Arase S. Angiostatic effects of corticosteroid on wound healing of the rabbit ear. J Med Invest. 2002;49(1–2):61–6. 11901762

[69] Hsu KC, Luan CW, Tsai YW. Review of Silicone Gel Sheeting and Silicone Gel for the Prevention of Hypertrophic Scars and Keloids. Wounds. 2017;29(5):154–8. 28570253

[70] O'Brien L, Jones DJ. Silicone gel sheeting for preventing and t reating hypertrophic and keloid scars. Cochrane Database Syst Rev. 2013(9):CD003826 doi: 10.1002/14651858.CD003826.pub3 24030657 10.1002/14651858.CD003826.pub3 PMC7156908

