# Supplementary figures and images for: Treatment of limb wounds of horses with orf virus IL-10 and VEGF-E accelerates resolution of exuberant granulation tissue, but does not prevent its development
Source: PLoS One. 2018 May 15;13(5):e0197223. doi: 10.1371/journal.pone.0197223 (PMC5953458; doi:10.1371/journal.pone.0197223)

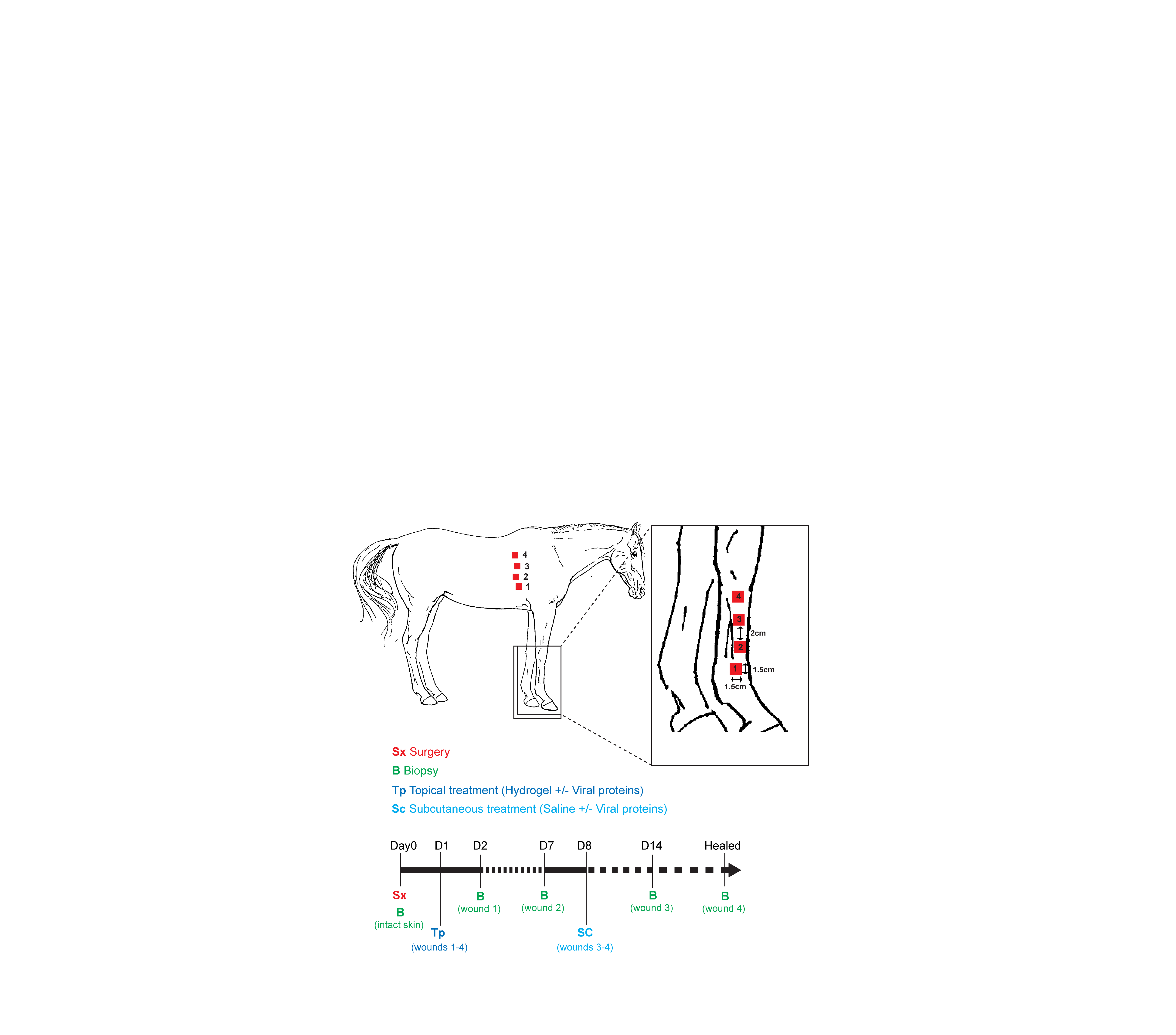

Supplement: S1 Fig — On day 0, each horse received four 1.5 cm X 1.5 cm (2.25 cm2) full-thickness skin wounds. After 24 hours, the wounds were administered hydrogel with or without the viral proteins (20μg VEGF-E and 2μg ovIL-10) to each of the four wounds of one randomly-assigned limb and one thoracic area. Limb wounds were bandaged for the duration of the study, while thoracic wounds were dressed following treatment for only 8–12 hours. After 8 days, the two wounds yet to be harvested received an additional treatment of saline with or without the viral proteins (10 μg VEGF-E and 1 μg ovIL-10) by subcutaneous injection. Biopsies were taken from one wound per site (treated body; control body; treated limb; control limb), in the order indicated (1, 2, 3, 4), on day 2, 7, 14 and once the wound had healed. (TIF) [file pone.0197223.s001.tif]

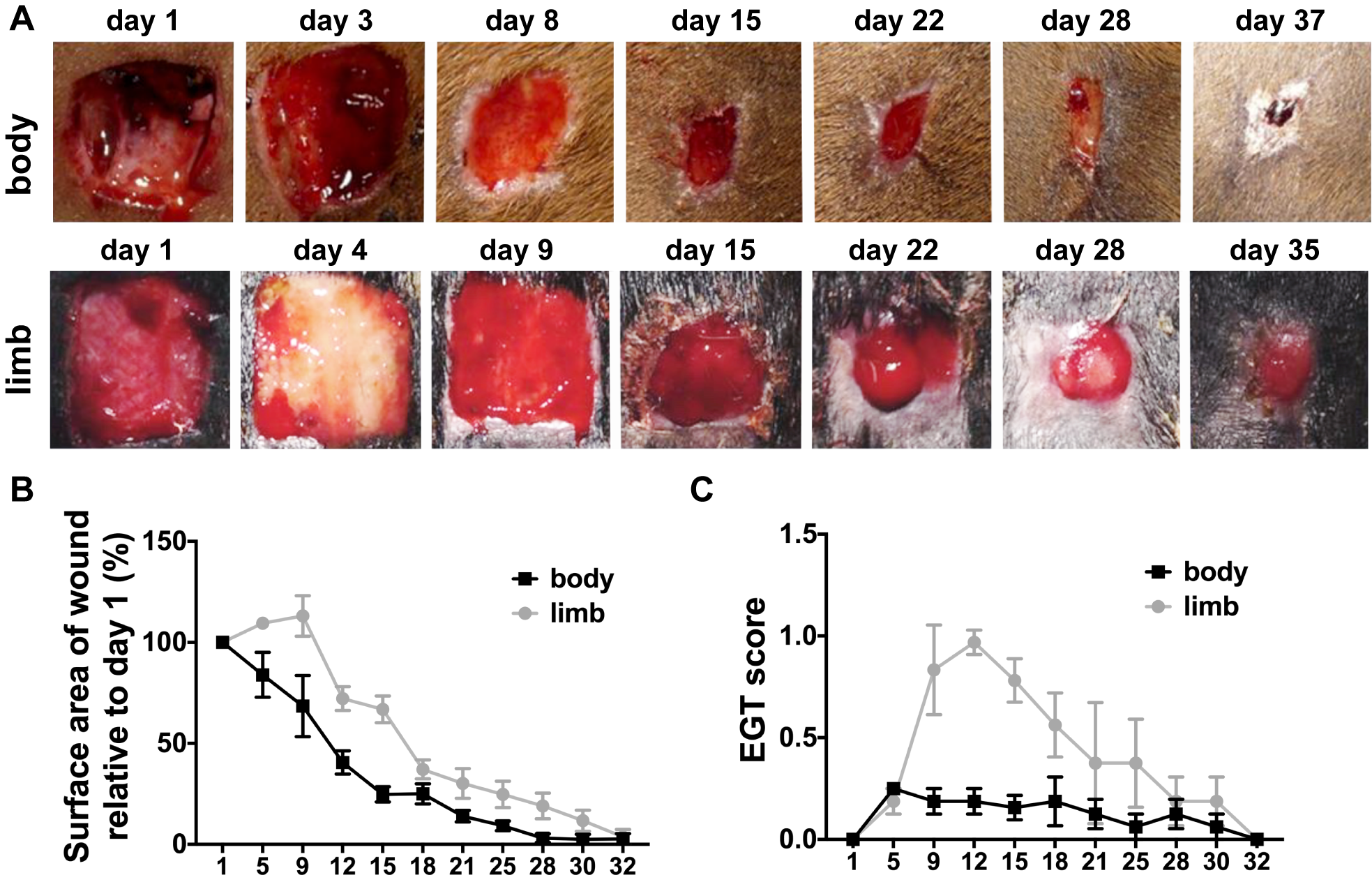

Supplement: S2 Fig — (A) Representative photos of healing wounds on the body (top row) or limb (bottom row) taken at the days indicated. (B) Surface area and (C) exuberant granulation tissue (EGT) formation of healing wounds at the days indicated. Wound surface area is calculated relative to the original wound area. EGT formation was scored 50% on protuberance (0 none– 2 marked), 25% on colour (0 pink– 1 yellow-red) and 25% on quality (0 smooth—1 rough). Values represent mean ± SEM, n = 4. (TIF) [file pone.0197223.s002.tif]
